# Sweeter and stronger: enhancing sweetness and stability of the single chain monellin MNEI through molecular design

**DOI:** 10.1038/srep34045

**Published:** 2016-09-23

**Authors:** Serena Leone, Andrea Pica, Antonello Merlino, Filomena Sannino, Piero Andrea Temussi, Delia Picone

**Affiliations:** 1 Dipartimento di Scienze Chimiche, Università di Napoli Federico II, I-80126, Napoli, Italy; 2 Department of Basic and Clinical Neurosciences, King’s College London, London SE5 9RX, UK

## Abstract

Sweet proteins are a family of proteins with no structure or sequence homology, able to elicit a sweet sensation in humans through their interaction with the dimeric T1R2-T1R3 sweet receptor. In particular, monellin and its single chain derivative (MNEI) are among the sweetest proteins known to men. Starting from a careful analysis of the surface electrostatic potentials, we have designed new mutants of MNEI with enhanced sweetness. Then, we have included in the most promising variant the stabilising mutation E23Q, obtaining a construct with enhanced performances, which combines extreme sweetness to high, pH-independent, thermal stability. The resulting mutant, with a sweetness threshold of only 0.28 mg/L (25 nM) is the strongest sweetener known to date. All the new proteins have been produced and purified and the structures of the most powerful mutants have been solved by X-ray crystallography. Docking studies have then confirmed the rationale of their interaction with the human sweet receptor, hinting at a previously unpredicted role of plasticity in said interaction.

Sweet proteins are a family of structurally unrelated proteins that can elicit a sweet sensation in humans. To date, eight sweet and sweet taste-modifying proteins have been identified: monellin^1^, thaumatin^2^, brazzein^3^, pentadin^4^, mabinlin^5^, miraculin^6^, neoculin^7^ and lysozyme^8^. With the sole exception of lysozyme, all sweet proteins have been purified from plants, but, besides this common feature, they share no structure or sequence homology^9^. Lately, sweet proteins have been receiving much attention, in response to the growing demand for new sugar replacers from food industry. Monellin, isolated from the African plant *Dioscoreophyllum cumminsii*, is, together with thaumatin, one of the most potent sweeteners known, being about 90,000 times sweeter than sucrose on a molar basis. Thanks to their incredible sweetness and non-carbohydrate nature, sweet proteins could ideally become the scaffold to build a new generation of high intensity sweeteners^10^, suitable also for people suffering from pathologies linked to glucose metabolism (*e.g.* diabetes, caries or hyperlipidaemia). The activity of sweet proteins depends on their three-dimensional structure, which, in turn, is sensitive to extreme physical parameters (temperature, pH or pressure), sometimes encountered during food processing. Protein engineering then becomes a valuable tool to improve sweet proteins’ performances, making them more suitable for industrial applications. In this framework, it is crucial to understand the structure/activity relationships of such molecules. All sweet proteins elicit a taste response upon interaction with the human taste receptor T1R2-T1R3, a heterodimeric G-protein coupled receptor (GPCR) located on specialised cells on the tongue^11^,^12^,^13^,^14^,^15^. This is the same receptor responsible for sensing all classes of sweet compounds, including sugars and small molecular weight sweeteners. Different sweet substances are recognised by different regions of the receptor^16^,^17^,^18^,^19^, but the large dimension of sweet proteins suggests an alternative mode of interaction. The proposed hypothesis to explain this phenomenon is the wedge model^17^,^20^,^21^, which suggests that, like other GPCRs, the sweet taste receptor exists in equilibrium between an active and a resting form; sweet proteins might stabilise the active form of the T1R2-T1R3 dimer by binding a wide cleft spanning both subunits of the receptor. Since complexes with sweet proteins have never been experimentally resolved, the wedge model has been built using a homology model of the receptor, based on the structure of the metabotropic glutamate receptor mGluR1^22^. Nonetheless, building on this model, it has been possible to rationalise the effects of point mutations affecting the potency of monellin, brazzein and thaumatin^23^,^24^,^25^. The widely accepted idea is that both proper surface charge distribution and three-dimensional shape have to be maintained in order to trigger the sweet sensation^23^,^25^,^26^,^27^,^28^. We have focused our attention on MNEI, a single chain derivative of monellin, a small (~11 KDa), globular protein. Wild type monellin has a cystatin-like fold, composed of two non-covalently linked chains^29^,^30^,^31^, which dissociate when heated above ~50 °C. This is accompanied by taste loss and prevents the use of the protein as a sweetener above this temperature. To circumvent this inconvenience, single chain derivatives with higher thermostability, among which MNEI, have been designed^31^,^32^. MNEI has the same sweetness as native monellin, with a recognition threshold of only 1.43 mg/L (127 nM)^33^ and a melting temperature of about 80 °C^34^,^35^. Nonetheless, even this protein can lose its sweetness if slight deformations of the three dimensional shape occur. For instance, mutation G16A, involving a buried residue of MNEI, only modifies the protein flexibility, but induces nearly complete loss of the sweet taste^36^,^37^,^38^.

The other factor that most significantly correlates with sweetness is surface charge: in fact, the surface of the T1R2-T1R3 complex that is described to bind sweet proteins is characterised by the presence of a large amount of acidic amino acids^17^,^21^. Studies on single and double chain monellins^23^,^28^,^39^,^40^, thaumatin^24^,^41^,^42^,^43^, brazzein^44^,^45^,^46^ and lysozyme^47^ have shown that, in general, mutations increasing the acidic character would consistently decrease or even cancel sweetness, whereas the outcome of the introduction of a positive charge is not immediately predictable. For instance, among four surface mutations, namely M42R, Y63R, Y65R and D68R, only Y65R would increase sweetness, whereas the other mutations, despite introducing a positive charge, would abate the taste intensity^23^. This is a consequence of the non-homogeneous charge distribution on the receptor surface, which implies that, in order to potentiate the effect of sweet proteins, positive charges have to be located in specific positions on their surface. Docking studies can only provide limited indications, as the structure of the receptor, built by homology, allows only low resolution predictions^17^,^21^. Recently, more advanced models have been built in terms of topological refinement^25^, by taking into account the information deriving from previous mutagenesis studies. These models have been able to account for many of the experimental outcomes of mutations of charged surface residues, and proved the possibility to predict at the atomic detail the complexes of mutants of MNEI and brazzein with the sweet receptor. More recently, a similar approach has been used to design and validate a new super-sweet mutant of thaumatin^24^. In the present study, we have started from the sweeter variant Y65R-MNEI^23^ and have introduced new, rationally predicted mutations to further potentiate sweetness. We then incorporated in the sequence of the sweetest construct mutation E23Q, which has been recently shown to increase stability at neutral to alkaline pH^35^. This latter mutation was not expected to affect MNEI sweetness, since the side chain of residue 23 is buried in a hydrophobic pocket and not exposed to interactions with the receptor. Surprisingly instead, it produced an additional gain in sweet taste, as proven by sensory evaluation, which ranked this construct as the sweetest protein ever produced. To elucidate details of their mode of action, the structure of the new mutants has been solved by X-ray crystallography and docked onto the sweet taste receptor. The results confirm the predictive value of the wedge model to design MNEI mutations and offer a new picture of the interaction between sweet proteins and the receptor.

## Results

### Design and characterisation of MNEI charge mutants

**Figure 1:**
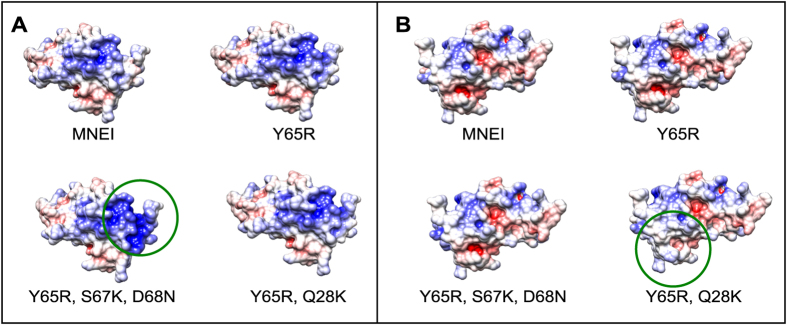
Comparison of the electrostatic potential maps of MNEI, Y65R-MNEI, C41S, Y65R, S67K, D68N-MNEI (Mut1) and Q28K, C41S, Y65R-MNEI (Mut2). Acidic (red) and basic (blue) regions are mapped to the molecular surface for the four constructs on the same (**A**) and opposite side (180° rotation, (**B**)) of mutation Y65R. Green circles highlight the regions where the mutations indicated in the labels were introduced to alter the surface charge distribution.

**Figure 2:**
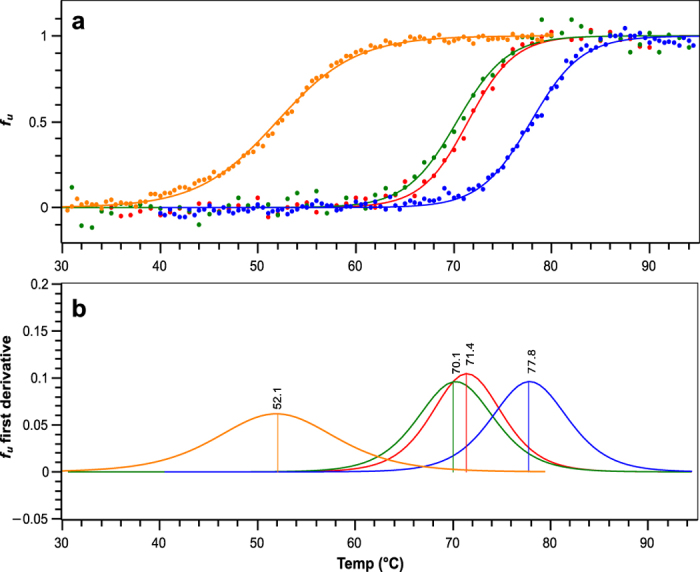
Thermal denaturation profiles of the different constructs. Comparison of CD unfolding curves (**a**) at pH 6.8 and their first derivatives (**b**) for Y65R-MNEI (red), Mut1 (orange), Mut2 (green) and Mut3 (blue). The image shows the differences in thermal stability produced by the various mutations introduced and highlights how mutation E23Q alone increases resistance to unfolding of about 8 °C.

In order to select the best possible mutations for new MNEI constructs, we analysed the electrostatic surface potentials of MNEI in comparison to a model of its sweeter and well characterised mutant Y65R-MNEI^23^, which we used as the starting point for the present mutagenesis experiments. Since residue 65 had previously been linked to sweetness enhancement, the surrounding region is likely involved in the interaction with the receptor. A comparison of the electrostatic potential maps for MNEI and Y65R-MNEI is presented in Fig. 1. We designed mutations localised in the same surface area of the protein, adding positive charge density on either the same or the opposite side of the surface with respect to R65. In the first case, we tested the additional mutations S67K and D68N, *i.e.* we added a basic residue and removed an acidic one, respectively (Fig. 1A). Residues S67 and D68 are both located on the loop connecting the β3 and β4 strands (*L34*) and were chosen since their mutation is not expected to significantly affect structural stability. Similar considerations were made when selecting mutation Q28K. In this case, with the introduction of a lysine residue, we aimed at reducing the negative charge density at the C-terminus of the helix, as evidenced in Fig. 1B. Mutation C41S was also introduced in both constructs. C41 is the only cysteine in the sequence and it is not involved in the formation of disulphide bridges, although it has been identified as the source of destabilisation of MNEI at extremely high pHs^48^. Mutation of C41 to serine was then introduced, despite previous studies on synthetic single chain monellin had correlated it to a minor decrease in sweetness^40^, to avoid undesired dimeric artefacts, which could arise from partial protein denaturation and oxidation. It is in fact known that both MNEI and Y65R-MNEI display a tendency to aggregate and form dimers and multimers, in particular at neutral to alkaline pHs^34^. The resulting constructs prepared for this study were therefore C41S, Y65R, S67K, D68N-MNEI (**Mut1**) and Q28K, C41S, Y65R-MNEI (**Mut2**). Both proteins were produced recombinantly in *Escherichia coli* BL21 and purified by ion exchange chromatography with slight modifications of previously published protocols^49^. Mut1 showed a marked tendency to precipitate during the purification, leading to significantly lower yields compared to Mut2. Nonetheless, both proteins could be obtained at high purity, and CD spectra were recorded. Comparison with Y65R-MNEI showed a nearly identical global fold and the persistence of the β-sheet rich structure typical of monellins (Supplementary Fig. S1). CD spectroscopy was also used to record thermal denaturation profiles (Fig. 2a). The experiments were performed at neutral pH, at which native monellin displays lower stability and higher propensity to aggregation^34^,^48^,^50^. Mut2 exhibited a stability comparable to that of Y65R-MNEI (T_m_ 70.1 and 71.4, respectively), whereas Mut1 appeared much less stable, with a Tm of 52.1 °C, about 20 °C lower than Mut2 and Y65R-MNEI (Fig. 2b). This destabilisation is probably the result of excessive positive charge density within a small surface area and could explain the solubility problems encountered in the purification of this mutant. Irrespective of its sweetness, the poor performance of Mut1 in terms of stability makes it an unlikely candidate for food and beverage applications, where high temperatures are often encountered. Mut2, on the other hand, appears more promising, to the point that we decided to incorporate in its sequence the stabilising mutation E23Q, which has been shown to remove the stability dependency from pH^35^. **Mut3** (E23Q, Q28K, C41S, Y65R-MNEI) was expressed and purified and its thermal denaturation profile was evaluated. As shown in Fig. 2, Mut3 is more resistant to thermal denaturation (T_m_ 77.8 °C) than Y65R-MNEI and Mut2, thus seeming a more appealing candidate for the development of industrial applications.

### Sensory evaluation of the sweet proteins

**Figure 3:**
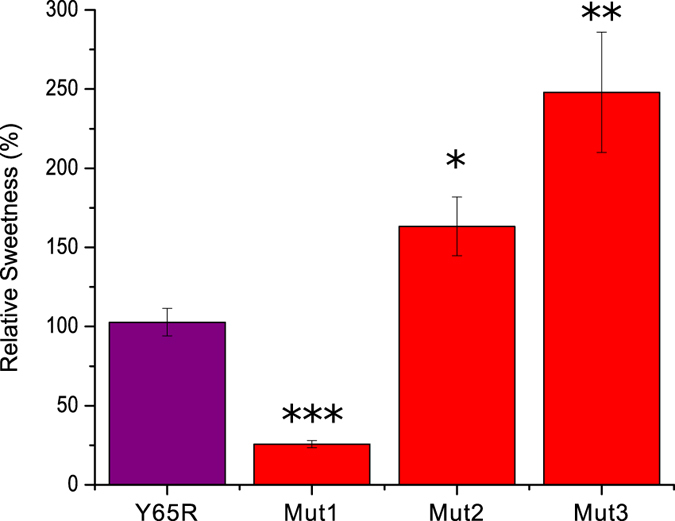
Sweetness threshold of the different constructs. Relative sweetness thresholds compared to Y65R-MNEI. (Mean ± SEM, *p < 0.02; **p < 0.01; ***p < 0.0001). Both Mut2 and Mut3 exhibit stronger sweetness compared to the control protein, whereas Mut1 possesses much weaker activity.

In order to assess the validity of the design in terms of sweetness improvement, all proteins were subjected to taste assessment. Relative sweetness was compared to that of Y65R-MNEI, and is reported in Fig. 3. Both Mut2 and Mut3 resulted sweeter than Y65R-MNEI. Sweetness thresholds were evaluated by triangle test by a panel of tasters, and resulted 2.50, 0.40 and 0.28 mg/L (223, 36 and 25 nM) for Mut1, Mut2 and Mut3, respectively. In comparison, Y65R-MNEI exhibited a threshold of 0.62 mg/L (55 nM), in agreement with previous results^34^. Surprisingly, although containing the same surface mutations, Mut3 and Mut2 showed different sweetness thresholds, suggesting that the stabilisation of the structure at neutral pH might play a role in the interaction of the mutants with the receptor.

### Structural characterisation of Mut2 and Mut3

**Figure 4:**
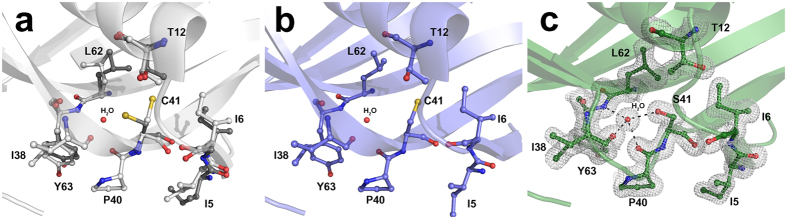
Detail of C41 mutation site. Residue C41 in the structures of MNEI deposited under PDB code 2O9U (**a**) and under PDB code 1IV7 (**b**) respectively. Residue S41 in the structure of Mut2 (**c**). 2F_o_-F_c_ electron density map is contoured at 1.0 σ.

**Figure 5:**
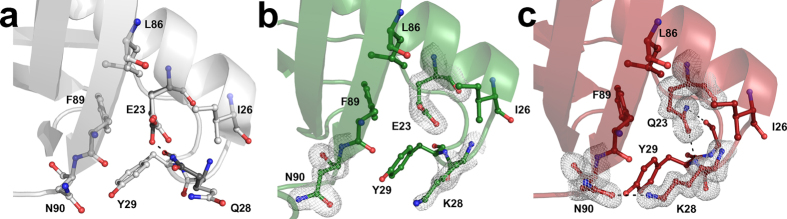
E23-Q28 mutation sites. Residue Q28 in the structure of MNEI deposited under PDB code 2O9U adopts two alternative conformations one of which is in direct contact with the side chain of residue E23 that is buried in a hydrophobic cavity formed by residues I26, Y29, L86 and F89 (**a**). In Mut2, upon Q28K mutation, this interaction is lost (**b**). In Mut3, E23Q mutation allows a rearrangement of K28, whose side chain forms an additional H-bond with N90 (**c**). The 2F_o_-F_c_ electron density maps are contoured at 1.0 σ.

In the attempt to rationalise the differences in term of taste potency between Mut2 and Mut3, we solved the structure of the two proteins by X-ray crystallography. Mut2 and Mut3 were crystallised under the same experimental conditions, resulting in two different space groups (Supplementary Table S1). For both mutants, the asymmetric unit (a.u.) contains two protein molecules strongly interacting with each other and resulting in a dimer. It is worth noting that MNEI can form crystals containing either a monomer (PDB code 2O9U)^51^ or a dimer (PDB code 1IV7) in the a.u., depending on the crystallisation conditions. Both the electron-density maps of Mut2 and Mut3 structures are very well defined, with the only exception of loop *L23* (residues 47–56) connecting strands *β2* and *β3*. This loop is usually highly flexible or disordered in the structures of MNEI and its derivatives. Therefore, these residues were excluded from any comparative analyses and from root mean square deviation (RMSD) calculation among structures. In both Mut2 and Mut3, mutations do not alter the overall protein fold: the structure is very similar to that of MNEI. RMSDs between main chain atoms of Mut2 and Mut3 in comparison to the reference structure for MNEI (PDB code 2O9U) are reported in Table 1. Mutation sites of Mut2 and Mut3 were analysed by visual inspection of the structures. In MNEI, C41 is located in a hydrophobic region lined by side chains of residues I5, I6, T12 and L62. In the X-ray structure of MNEI solved at atomic resolution (PDB code 2O9U), the side chain of C41 adopts two different conformations with occupancy 0.3 and 0.7, hereafter referred to as 1 (χ = −82°) and 2 (χ = −171°), respectively (Fig. 4a). In structure 1IV7, instead, the side chain of C41 adopts only conformation 2 (Fig. 4b). On the contrary, in both molecules present in the a. u. of Mut2 and Mut3, S41 side chain adopts conformation 1 and forms a hydrogen bond with a water molecule that connects S41 to main chain atoms of residues P40, I38, Y63 (Fig. 4c and Supplementary Fig. S2). Y65 is located on the surface of MNEI. The mutation Y65R introduces at this site a charged residue, whose side chain is highly flexible and explores different conformations in the structures of Mut2 and Mut3, forming several interactions with solvent molecules, sometimes involved in packing contacts (Supplementary Fig. S3). In the wild-type protein, Q28 is a solvent exposed residue; its side chain adopts two distinct conformations, one of which is in direct contact with the side chain of residue E23 that is buried in a hydrophobic cavity formed by residues I26, Y29, L86 and F89 (Fig. 5a). In Mut2, containing the mutation Q28K, the lysine side chain is pushed away toward the solvent, forming a stabilising interaction with the hydroxyl group of Y47 from a symmetry related mate in one of the two molecules present in the a.u. and remaining disordered in the other one (Fig. 5b). The introduction of the additional mutation E23Q significantly alters the structure of the surrounding residues: in Mut3, in fact, Q23 assumes a different conformation compared to E23 in both Mut2 and in MNEI and establishes new hydrogen bonds with the main chain atoms of Y29 and G30. The conformational variation of the side chain of Q23, when compared to E23, allows the rearrangement of the side chain of K28, which forms stabilising hydrogen bonds with main chain and side chain atoms of N90 (Fig. 5c).

### Interaction with the sweet taste receptor

**Figure 6:**
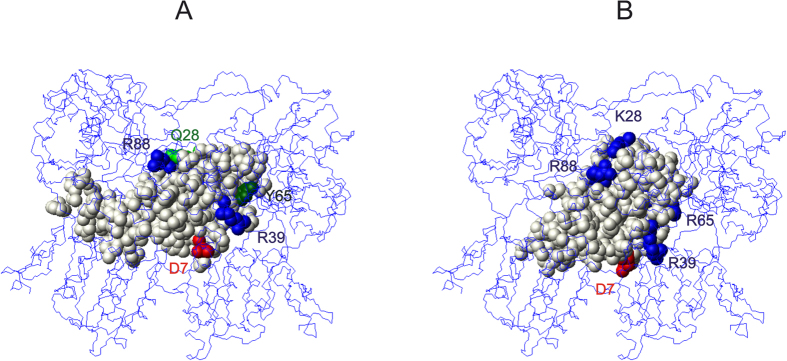
Comparison of the interaction of MNEI and Mut3 with the sweet receptor. Molecular models of the sweet proteins are represented as full atom; the receptor is represented as a line model. (**A**) Interaction surface of MNEI with the sweet receptor; basic residues are represented as blue balls, acidic residues as red balls, Q28 as green balls, Y65 as dark green balls. (**B**) Interaction surface of Mut3 with the sweet receptor; basic residues are represented as blue balls, acidic residues as red balls.

The interaction of MNEI with the T1R2-T1R3 has been interpreted in the framework of the first mechanism proposed for the interaction of the three sweetest natural proteins, the so-called wedge model^17^,^21^. Although this model is still the only general model for the interpretation of the interaction of sweet proteins with the receptor, it has been the subject of some criticism. For instance, Assadi-Porter *et al*. claimed that receptor mutations based on the wedge model did not suppress the interaction of brazzein with the receptor^44^. However, the failure to predict correct mutations in the receptor, was due to the blind use of the model^25^. In its simplest formulation, in fact, the wedge model only yields an ensemble of protein molecules that bind with different orientations and even with slightly different parts of their surface^25^ and unsuccessful predictions based on the model were obtained when a single orientation was arbitrarily chosen^44^. By using a tethered docking approach, we were able to show that topologically correct models of complexes of monellin and brazzein with the sweet receptor are indeed consistent with the distribution of charged residues and explain data not used in their initial derivation^25^. Recent experimental work from Assadi-Porter *et al*. indeed confirmed the coherence of the revised model with their observations on new brazzein mutants^52^. Accordingly, we decided to use the wedge model to interpret the huge increase in sweetness observed in Mut3. When checking the consistency of the new mutations, which lead to a protein even sweeter than Y65R-MNEI, and particularly the crucial Q28K mutation, it was natural to try to align the X-ray structure of Mut3 with the structure of MNEI in the topological complex^25^. Although the conformations of side chains are inevitably different, most of the charged residues previously selected for the tethered docking are still at distances compatible with good electrostatic interaction with receptor residues of opposite charges. However, the side chain of K28 is far from the interface between MNEI and the receptor. This result is apparently inconsistent with the wedge model: it can be explained by accepting either that the complex generated by the mentioned tethered docking is inaccurate, or by hypothesising that it is possible to have multiple interaction surfaces. We checked this possibility by first trying to bring the side chain of K28 closer to the receptor and then mapping the new interaction interface. It was soon clear that a simple rotation of ca. 30° along the long axis of the molecule of Mut3, in the orientation consistent with the model complex of MNEI^21^ was all that was needed. What came as a big surprise was the permanence of several interactions, notably those involving D7, R39, R88 and R65. In other words, it appears that these crucial residues are in a pivotal position with respect to the mentioned rotation. This result was double-checked using the tethered docking approach previously used to refine the complex of MNEI^21^. After minor adjustments we found that the main contacts between Mut3 and receptor residues can be summarised as follows: the C_γ_ atom of D7 of Mut3 is at 3.3 Å from the C_ζ_ atom of R247 of T1R3; likewise C_ζ_ of R39 is 7.1 Å from C_β_ of D169 of T1R2, C_ζ_ of R88 is 3.46 Å from C_γ_ of E47 of T1R3, Cε of K28 is 5.0 Å from C_γ_ of E48 of T1R3 and C_ζ_ of R65 is 4.23 Å from C_β_ of D456 of T1R2. The relationship between the two interacting surfaces is illustrated in Fig. 6. The resolution of the docking model allows detecting the contact points between the sweet protein and T1R2-T1R3, but is unfortunately not sufficient to individuate the subtle differences in closely related proteins such as Mut2 and Mut3, which translate in the different biological activity.

## Discussion

Changes in dietary habits have led to an increase in pathologies related to carbohydrates metabolism, such as obesity, diabetes, hyperlipidaemia and caries, with repercussions on life style and health care costs. Food and beverage industries are in constant search of new sweetening compounds, whose ideal characteristics would be safety and palatability. Sweet proteins represent a potential resource in this respect: their proteinaceous nature hints at safety, their amazing sweetening power allows for the use of minimum quantities and the possibility of obtaining them through recombinant technologies opens the way to large scale production^10^,^53^. Moreover, protein design can help to improve their characteristics, tuning their performance in view of real life applications. We have used this approach to enhance sweetness and resistance to thermal denaturation and pH variations of MNEI, a single chain monellin, as these features are of primary importance for applications to food and beverages. Starting from the well characterised mutant Y65R-MNEI^23^,^34^ and based on the prediction of the surface of interaction with the T1R2-T1R3 sweet receptor, we have designed two different charge mutants. One of them, Mut1 (C41S,Y65R,S67K,D68N-MNEI), exhibited a drop in sweetness, despite presenting an increased positive surface charge. The other construct, Mut2 (Q28K,C41S,Y65R-MNEI), displayed instead amazing potency, with a recognition threshold of only 36 nM, roughly 30% lower than Y65R-MNEI and 3.5 times sweeter than the parent protein, according to literature data for MNEI^34^. These results underline the importance, for sweet proteins, to present the correct pattern of positive charges on the surface of interaction with the receptor and is in line with the outcome of previous mutagenesis studies^23^,^28^,^41^,^42^. Although recombinant production of the sweet taste receptor has been achieved^14^,^44^ and experimental mapping of the interactions between sweet proteins and their receptor would at this point be feasible, a similar approach is very time and resources consuming. Instead, our results support the validity of the wedge model in predicting and explaining the physiological effects of mutated sweet proteins, confirming its applicability to drive *in silico* sweet proteins’ design.

Among the designed constructs, Mut1 was less thermally stable than the reference protein Y65R-MNEI. Since stability towards pH and temperature variations is indeed a desirable attribute for a protein with potential applications to large scale processes, we designed Mut3, with the same sequence of Mut2 and the additional stabilising mutation E23Q^35^. The increased stability introduced by this mutation had been linked to the formation of hydrogen bonds between the side chain of Q23 and the backbone atoms of Y29 and G30^35^. The crystal structures confirmed the existence of these contacts and highlighted additional stabilising interactions between the side chain of K28 and neighbour residues, triggered by the conformational change of the side chain of K28 induced by mutation E23Q, which further clarify the gain in thermal stability. In previous studies, residue E23 had been the target of several mutations, since this amino acid, located in a hydrophobic pocket of MNEI structure, has a crucial role in the stability of the protein. Mutations introducing hydrophobic residues at this position consistently increased thermal stability of MNEI^39^,^48^,^54^. In terms of biological activity, alanine replacement had no effect on sweetness^39^, whereas replacement with other hydrophobic amino acids, such as leucine, phenylalanine or tryptophan was accompanied by a slight flavour decrease compared to MNEI, despite helping to retain sweetness even after prolonged treatments at elevated temperatures^54^. These effects could be the result of minor modifications of the protein structure or flexibility, undetectable by the spectroscopic techniques (*i.e.*, CD) employed to characterise the constructs^39^,^54^. Mutation E23Q, which improves to the same extent MNEI thermal stability, has the opposite effect, resulting in a further decrease of the sweetness threshold, down to 25 nM, which makes of Mut3 the sweetest protein designed to date. Such exceptional sweetness, compared to Mut2, could be ascribed to various causes: the above described subtle structural differences around K28 may play a role also in defining the interaction with T1R2-T1R3 receptor. Moreover, small differences in flexibility between the two proteins could affect the binding to the receptor. The model based on tethered docking suggests for Mut3 a new interacting surface. Within the complex, all the pivotal interactions, previously detected in the topologically refined complex between MNEI and the receptor, are retained, but, in addition, the new interacting side can be obtained from that of MNEI^25^ by a rotation of ca. 30° along the longest protein axis. While further supporting the validity of the wedge model, these results present us for the first time with the idea that the interaction between sweet proteins and the receptor might be endowed with a certain plasticity: the possibility of multiple mutual orientations of the sweet protein and the active form of the receptor suggests that entropic factors might also be involved and play a determinant role, in providing sweet proteins with their extreme potency.

## Methods

### Structures and surface electrostatics

All mutants were designed using model 1 of PDB code 1FA3 as a template^55^. Protein secondary structure elements are referred to within the text using the following nomenclature: *Nt* (1–4), *β1* (4–6), *Lα1* (6–9), *α1* (10–27), *Lα2* (28–34), *β2* (35–48), *L23* (49–54), *β3* (55–64), *L34* (65–68), *β4* (69–78), *L45* (79–83), *β5* (84–90), *Ct* (91–96). Visualisation and manipulations of the structures were performed with UCSF Chimera^56^. Surface electrostatic calculations were performed with Delphi^57^, using a dielectric constant for the protein ε_P_ = 4 and a salt concentration of 150 mM.

### Sensory evaluation

Sweetness threshold was evaluated by triangle test as previously described^23^,^24^. MNEI solutions were used as positive control and water as negative control. Three paper cups, one containing 5 mL of protein solution and the others containing 5 mL of mineral water, were given to the panelists, who were asked to indicate which cup had the taste-eliciting solution and to rate the taste from 0 (no taste) to 5. A value of 1 indicated the perception of a taste, 2 meant the taste was recognised as sweet. The sample solutions were provided from the lowest (5 nM) to the highest (500 nM) concentration. The subjects tasted the compound without any time constrains, then spat it and rinsed with mineral water within a 1 min interval. Sweetness threshold was the concentration at which the protein scored 2 on average. Relative sweetness percentage = (MNEI-Y65R threshold/sample threshold) × 100. Data are presented as mean ± SEM; the significance of differences of the mean values was confirmed by post hoc analysis of variance (OriginLab one-way ANOVA routine). Global analysis with Bonferroni correction yielded *p* < 0.0001.

### Expression and purification of the mutants

Synthetic genes encoding for the sequence of Mut1, Mut2 and Mut3 were purchased from Eurofins Genomic. The genes were cloned in the pET22b(+) expression vector between the *NdeI* and *SacI* sites. Protein were expressed in *Escherichia coli* BL21(DE3) and purified from the cell lysate by a coupled anion/cation exchange procedure as previously described^49^.

### Circular Dichroism Spectroscopy

Circular dichroism (CD) spectra were recorded on a Jasco J-715 spectropolarimeter equipped with a Peltier temperature control system (PTC-348WI). Molar ellipticity per mean residue [θ] in deg cm^2^ dmol^−1^ was calculated from the equation: [θ] = [θ]_obs_ mrw/(10 × l × C), where [θ]_obs_ is the ellipticity measured in degrees, mrw is the mean residue molecular weight of the protein (Da), C is the protein concentration in g/mL and l is the optical path length of the cell in cm. Cells of 0.1 cm path length were used. CD spectra were recorded with a time constant of 4s, a 2 nm band width and a scan rate of 20 nm/min, and the signal was averaged over three scans and baseline corrected by subtracting the buffer spectrum. Spectra were recorded in 20 mM phosphate buffer pH 6.8 at a concentration of 0.2 mg/mL protein, as determined by UV absorbance at 280 nm.

Thermal denaturation experiments were recorded following the signal at 215 nm while varying the temperature from 30 to 95 °C at a rate of 1 °C/min. For each condition, three independent measures were performed. Experimental points were fitted to a Boltzmann curve, and fraction of unfolded protein (*f*_*u*_) was calculated according to the formula (1):


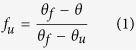


where θ_f_ and θ_u_ are the CD signal of the folded and unfolded state from the fitted curve, respectively, and θ is the CD signal at each temperature.

### Crystallisation and structure determination

Mut2 and Mut3 were dissolved in 10 mM HCl up to a concentration of 5.0 mg/mL. Crystals of both mutants were obtained at 20 °C using hanging-drop vapour-diffusion method and mixing an equal volume of protein and of a reservoir solution containing 30% PEG4K, 0.1 M sodium acetate at pH 4.6 and 0.2 M ammonium sulphate. X-ray diffraction data were collected at XRD1 beamline of Elettra Synchrotron (Trieste, Italy), using a detector Pilatus-6M (Dectris) and the wavelength of 1.065 Å. Before being exposed to the X-ray beam, the crystals were soaked into a cryo-solution consisting of mother liquor added of 30% glycerol and flash cooled in liquid nitrogen. Data sets were indexed, integrated, reduced and scaled using XDS and SCALA^58^. Data collection statistics are reported in Supplementary Table S1. The structures of Mut2 and Mut3 were solved by molecular replacement using the program Phaser^59^ and the structure of MNEI, without water and ligands, as the search model (PDB code 2O9U)^51^. Structures of the mutants were improved by iterative cycles of manual fitting using Coot^60^ and were refined by REFMAC5^61^ and Phenix^62^. 5% of the data was used for calculation of the R-free value. The structure of Mut2 was refined at 1.70 Å resolution to an R-factor of 18.8% (R-free 22.9%); the structure of Mut3 was refined at 1.55 Å resolution to a R-factor of 19.8% (R-free 23.9%). 98.2% and 1.8% residues in Mut 2 and 97.7% and 2.3% residues in Mut3 are located in the most favourable and allowed regions of the Ramachandran plot, respectively Refinement statistics are reported in Supplementary Table S1. Final coordinates and structure factors were deposited in the Protein Data Bank under the accession code 5LC6 for Mut2 and 5LC7 for Mut3.

### Complex refinement

The first ensembles of complexes of MNEI with different models of the T1R2-T1R3 receptor were built using the GRAMM software in a low resolution mode^63^. A single topological model of the complex was later obtained^25^ using GRAMM-X, a version of GRAMM accessible in Internet (http://vakser.bioinformatics.ku.edu).

In the web version of GRAMM it is possible to add residues suggested by the low-resolution ensemble that may belong to the interface of the complex and hypothetical residues suggested from mutagenesis data. Altogether, we favoured charged residues, mainly because of the mentioned importance of electrostatic interactions in the wedge model. The following residues were selected: (T1R2)_ D169, E170, R172, D173, K174, R176, D213, R217, D218, D456, R457, and (T1R3)_ R177, D190, R191, D216 and key charged residues of Mut3, *i.e.* D7, K28, R39, R65 and R88. Other parameters were maximised as described before^25^.

## Additional Information

****Accession codes:**** The crystal structures for Mut2 and Mut3 are deposited in the Protein Data Bank with accession codes 5LC6 and 5LC7, respectively.

**How to cite this article**: Leone, S. *et al*. Sweeter and stronger: enhancing sweetness and stability of the single chain monellin MNEI through molecular design. *Sci. Rep.* **6**, 34045; doi: 10.1038/srep34045 (2016).

## Supplementary Material

Supplementary Information

## Figures and Tables

**Table 1:** Main chain atom rmsd (Å) between the mutants and MNEI.

	MNEI	Mut2	Mut3
**MNEI**	—	0.62/0.66	0.59/0.72
**Mut2**	—	0.47*	0.64/0.50**0.50/0.54***
**Mut3**		—	0.56*

Comparison of the structural data between the mutants and MNEI (PDB code 1O9U).

^*^The number refers to the superposition of the two molecules in the asymmetric unit.

^**^The numbers refer to the superposition of one molecule of Mut2 on the two molecules of Mut3 in the asymmetric unit.

^***^The numbers refer to the superposition of the other independent molecule of Mut2 on the two molecules of Mut3 in the asymmetric unit.
