# Supplementary material for: Sweeter and stronger: enhancing sweetness and stability of the single chain monellin MNEI through molecular design
Source: Sci Rep. 2016 Sep 23;6:34045. doi: 10.1038/srep34045 (PMC5034325; doi:10.1038/srep34045)
Supplement: Supplementary Information [file srep34045-s1.pdf]

Sweeter and stronger: enhancing sweetness and stability of the single chain monellin MNEI through molecular design

Serena Leone, Andrea Pica, Antonello Merlino, Filomena Sannino, Piero Andrea Temussi and Delia Picone

Supplementary Information

Figure S1. Comparison of the CD spectra of the purified mutants. Mean residue ellipticity (MRE)of Y65R-MNEI (red), Mut1 (orange), Mut2 (green) and Mut3 (blue). CD spectra indicate that all proteins possess a high beta sheet content, a feature typical of the cystatin fold.

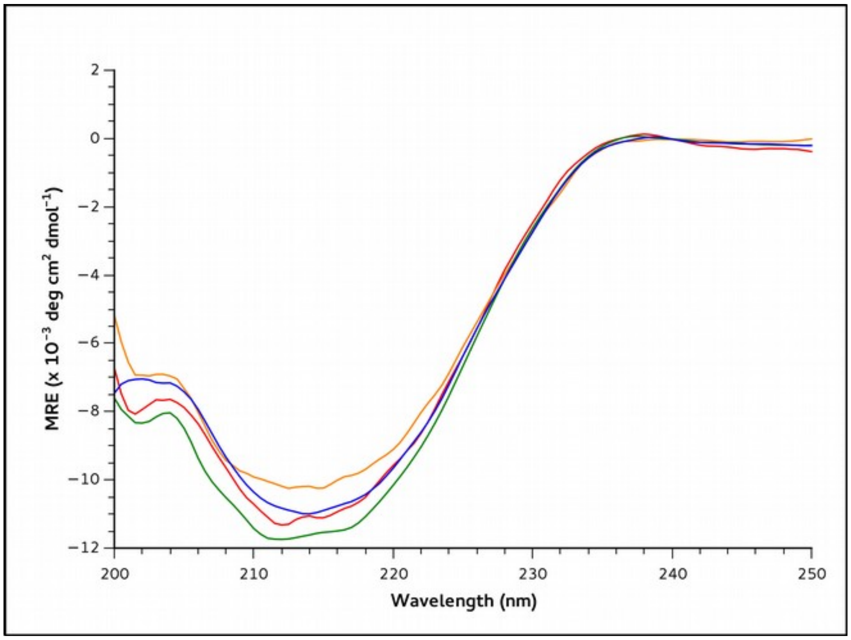

Figure S2. C41 mutation site. F_o-F_c electron density map calculated using Mut2 structure factors and phases from the structure of Mut2 with a cysteine in position 41. The negative peak, colored in red, indicates that this residue has been mutated in a serine. The map is contoured at 9.0 σ.

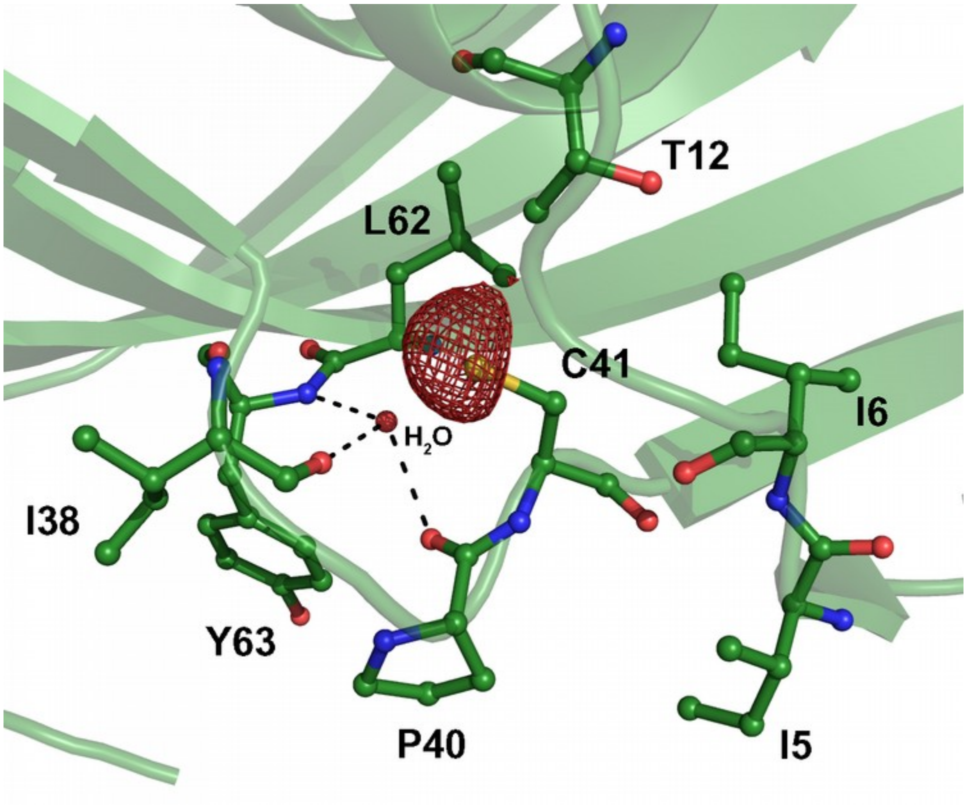

Figure S3. Y65R mutation sites. $2F_o$-F_c electron density maps for R65 in both molecules in the asymmetric unit of Mut2 and Mut3 are contoured at 1.0 σ

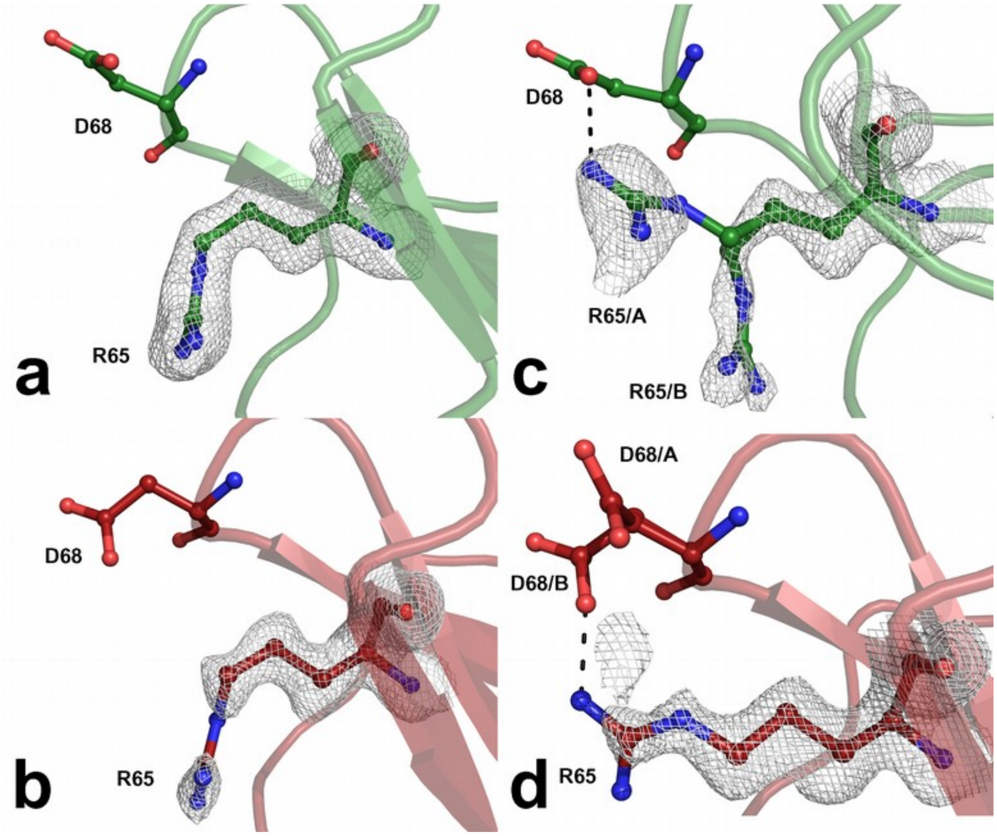

Table S1. Data collection and diffraction statistics. Numbers in parentheses refer to the highest resolution shell.

	**Mut2**	**Mut3**
**Data Collection**		
Crystallization conditions	30% PEG4K<br>0.1 M CH_3OONa<br>0.2 M $(NH_4)_2SO_4$	30% PEG4K<br>0.1 M CH_3OONa<br>0.2 M $(NH_4)_2SO_4$
pH	4.6	4.6
Space group	C2	P1
Cell dimensions		
a, b, c (Å)	129.7, 31.0, 45.5	31.5, 39.3, 44.0
α, β, γ (°)	90.0, 106.2, 90.0	105.3, 109.1, 104.0
Resolution limits (Å)	50.00 - 1.70<br>(1.74 - 1.70)	50.00 - 1.55<br>(1.59 - 1.55)
No. of observations	72221 (5545)	57157 (3853)
No. of unique reflections	18905 (1406)	23642 (1591)
Completeness (%)	96.9 (97.2)	90.2 (82.6)
I / σI	12.7 (1.5)	11.4 (2.3)
Redundancy	3.8 (3.9)	2.4 (2.4)
$R_{merge}^{\dagger}$ (%)	6.6 (91.0)	5.3 (42.5)
R_{meas} (%)	7.6 (105.2)	6.7 (53.9)
$CC_{1/2}$	99.8 (73.0)	99.7 (82.4)
**Refinement**		
Resolution (Å)	50.0 – 1.70<br>(1.74 – 1.70)	50.0 – 1-55<br>(1.59 – 1.55)
No. reflections	17877 (1204)	22141 (1548)
R_{work} / R_{free} (%)	18.8 / 22.9	19.8 / 23.9
No. atoms	1665	1706
Protein	1526	1555
Ligand/Ion	20	-
Water	119	151
Bfactors	23.7	18.2
Protein	22.8	17.1
Ligand/Ion	34.2	-
Water	33.8	28.9
R.m.s. deviations		
Bond lengths (Å)	0.019	0.021
Bond angles (°)	0.009	1.933
